# Evaluation of electrolyzed water to control fungal trunk pathogens in grapevine nurseries

**DOI:** 10.1002/ps.8568

**Published:** 2024-12-03

**Authors:** Mónica Berbegal, Adolfo Blasco, Grégoire Gaume, Pedro Amorós, Antônia Fernandes, José V. Ros‐Lis, Josep Armengol

**Affiliations:** ^1^ Instituto Agroforestal Mediterráneo Universitat Politècnica de València Valencia Spain; ^2^ REDOLí research group, Instituto Interuniversitario de Investigación de Reconocimiento Molecular y Desarrollo Tecnológico (IDM) Universitat Politècnica de València, Universitat de València Burjassot Spain; ^3^ Aquactiva Solutions SL Valencia Spain; ^4^ Institut de Ciència dels Materials (ICMUV) Universitat de València Paterna Spain

**Keywords:** Botryosphaeria dieback, black foot, grapevine propagation process, pathogen control, Petri disease, *Vitis vinifera*

## Abstract

**BACKGROUND:**

Grapevine producers demand solutions to control fungal trunk pathogens (FTPs) in nurseries. Adopting integrated strategies combining several control methods has been indicated as the best approach to prevent or reduce infections on grapevine propagation material. In recent years, electrolyzed water (EW) has emerged as a sustainable alternative for disinfection. Thus, the objectives of our study were: (i) to determine the effect of EW on the conidial germination and mycelial growth of a wider selection of FTPs associated with different grapevine trunk diseases; and (ii) to evaluate the efficacy of EW to reduce infections caused by FTPs on grapevine planting material during the propagation process in a commercial nursery.

**RESULTS:**

*In vitro* experiments demonstrated the capacity of different EW products to reduce conidial germination and mycelium survival of selected FTPs belonging to different genera and species, even given that the results were variable depending on the type of product, pathogen evaluated and time of treatment. In two different nursery experiments, conducted in 2021 and 2023, EW‐treated plants showed lower incidence of Petri and black‐foot associated pathogens when compared with the untreated ones, although these differences were statistically significant only in 2023. Moreover, there were no negative effects of the EW treatments regarding the viability of the grafted plants.

**CONCLUSIONS:**

Our results about the effect of EW against conidia germination and mycelium survival of FTPs, and the results of the nursery trials, suggest that EW could have promising applications in the grapevine nursery process. This treatment could be integrated with other complementary management strategies and also be extended to nurseries of other fruit and nut crops, in which FTPs are currently becoming important emerging diseases. © 2024 The Author(s). *Pest Management Science* published by John Wiley & Sons Ltd on behalf of Society of Chemical Industry.

## Graphical abstract

Electrolyzed water treatments could have promising applications in the grapevine nursery process to prevent or reduce infections caused by fungal trunk pathogens on grapevine propagation material.

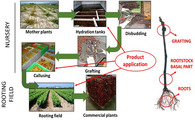

## 1 INTRODUCTION

Increasing incidence of diseases caused by fungal trunk pathogens (FTPs), which cause important production losses, have been reported on fruit crops such as pome, stone fruit, nut, berry fruit, citrus, grapevine and olive.^1^ Amongst them, the situation is especially serious in the case of grapevine, in which FTPs have caused untenable economic losses to the wine and table grape industry worldwide since the 1990s.^2^, ^3^, ^4^

Grapevine producers demand solutions to control FTPs in both grapevine nurseries and vineyards. Special focus has been placed on the development of procedures and products to prevent or reduce infections caused by FTPs on grapevine propagation material, because vineyards planted with fungal‐infected material often result in a high percentage of declining plants with poor vine vigor. Internally, these plants exhibit black discoloration and brown‐to‐dark streaks in the xylem, or sectorial necrosis of woody tissues. Consequently, growers are forced to replant sizeable vineyard areas.^2^, ^4^ However, it is not easy for nurseries to ensure a FTP‐free stock because during the grapevine propagation process there are many opportunities for infection.^2^, ^4^ There have been advances in the development of chemical, physical and biological control, and other management strategies to prevent or reduce infection of woody tissues by FTPs.^4^, ^5^, ^6^, ^7^, ^8^ Nevertheless, the scarcity of efficient active ingredients, complexity of the diseases, and the high infection risks, suggest that adopting integrated strategies combining several of these control methods rather than single solutions will be the best approach.^5^, ^6^, ^8^

In recent years, electrolyzed water (EW) has emerged as a sustainable alternative for disinfection because it is generated from water, salt (NaCl) and electricity through electrolysis. The electrochemical oxidation of chloride generates chlorine gas (Cl_2_) at the anode that dismutes generating hydrochloric and hypochlorous acids (HCl and HClO). The technologies of EW generators vary depending on the existence or not of a membrane separating the cathode and the anode, which prevents the mixing of the two solutions. A second variation is the recirculation of part of the solution generated at the cathode back into the cell. The combination of these two approaches can result in an EW with variable pH, between neutral and slightly acidic. The resulting neutral or slightly acidic EW is less corrosive and therefore more suitable for most applications.^9^, ^10^

Electrolyzed water has been described mainly as a biocide.^10^ EW activity includes antibiofilm properties,^11^ disinfectant of surfaces or air,^12^, ^13^ and shelf‐life promoter for various food products such as fruits and vegetables.^14^ Food industry pathogens for which EW has been successfully applied include *Campylobacter*,^15^ *Escherichia coli* ^16^ and *Salmonella*.^17^ Moreover, its application to control mycotoxins^18^ or for the disinfection of animal farms^19^ also has been explored.

There is less information about the application of EW against fungal pathogens. EW has been used to eliminate fungal spores from tropical fruits with ≤100% inactivation.^20^ Concentrations of Cl inhibited the growth of *Botrytis cinerea* showing significant curative effects.^21^ Moreover, its fungicide efficacy against powdery mildew (*Podosphaera cerasi*) on sweet cherry trees (*Prunus avium*),^22^ and *Penicillium* spp. in citrus^23^, ^24^ and other molds in celery and cilantro,^25^ has been demonstrated.

EW has been only barely explored for the control of grapevine pathogens. Giacosa *et al*.^26^ evaluated the use of EW in postharvest treatments of grapes for winemaking, and Magistà *et al*.^27^ investigated the efficacy of EW as a substitute for fungicides to reduce the incidence of *Aspergillus carbonarius* ochratoxin A contamination on grapes. EW activity to avoid microbial spoilage in wine was evaluated by Rego *et al*.^28^ Regarding FTPs, Di Marco *et al*.^29^ studied the effect of acid EW (pH 2.5–3.1) on the conidial germination and mycelial growth of *Phaeoacremonium minimum* and *Phaeomoniella chlamydospora*, and on grafted vines previously inoculated with *Pa*. *chlamydospora*. The acid EW reduced conidial germination in both pathogens. Moreover, nursery experiments revealed no negative effect in the growth of plants and a remarkable reduction of the infection level in the treated plants. However, this research was limited to only some of the causal agents of Petri and esca diseases of grapevines.

Thus, the objectives of our study were: (i) to determine the effect of EW on the conidial germination and mycelial growth of a wider selection of FTPs associated with different grapevine trunk diseases; and (ii) to evaluate the efficacy of EW to reduce infections caused by FTPs on grapevine planting material during the propagation process in a commercial nursery.

## 2 MATERIALS AND METHODS

### 2.1 Fungal isolates

In this study, we used nine isolates of the following species of grapevine FTPs: *Botryosphaeria dothidea*, *Cadophora luteo‐olivacea*, *Dactylonectria torresensis*, *Eutypa lata*, *Ilyonectria liriodendri*, *Lasiodiplodia theobromae*, *Neofusicoccum parvum*, *Pm. minimum* and *Pa. chlamydospora* (Table 1). These isolates were obtained from grapevines showing internal symptoms of wood necrosis or black vascular streaks. They were single‐spored or hyphal‐tipped before storage in 15% glycerol solution at −80 °C into 1.5‐mL cryovials in the fungal collection at the Instituto Agroforestal Mediterráneo, Universitat Politècnica de València, Spain.

**Table 1:** Isolates of grapevine fungal trunk pathogens and their associated diseases in nurseries used in the conidial germination and mycelial growth experiments

	Code	Grapevine disease	Location	Conidial germination	Mycelial growth
*Botryosphaeria dothidea*	GIHF‐158	Botryosphaeria dieback	Requena (Valencia)		+
*Cadophora luteo‐olivacea*	GIHF‐240	Petri disease	Aielo de Malferit (Valencia)	+	
*Dactylonectria torresensis*	GIHF‐154	Black‐foot	Requena (Valencia)	+	+
*Eutypa lata*	DT‐103	Eutypa dieback	Albacete (Albacete)		+
*Ilyonectria liriodendri*	GIHF‐363	Black‐foot	Llanera de Ranes (Valencia)	+	+
*Lasiodiplodia theobromae*	GIHF‐272	Botryosphaeria dieback	Sant Sadurní d'Anoia (Barcelona)		+
*Neofusicoccum parvum*	GIHF‐271	Botryosphaeria dieback	Sant Sadurní d'Anoia (Barcelona)		+
*Phaeoacremonium minimum*	GIHF‐098	Petri disease	Argamasilla de Alba (Ciudad Real)	+	+
*Phaeomoniella chlamydospora*	GIHF‐101	Petri disease	Sinarcas (Valencia)	+	+

*Note*: + Indicates in which type of experiment each isolate was used.

### 2.2 EW products

Experiments were conducted using different freshly prepared EW products, which were produced from a generator of EW (ELA‐200) (Aquactiva Solutions, Valencia, Spain) departing from deionized water and high‐quality NaCl (>99%; Sigma‐Aldrich, St Louis, MO, USA). The EW generator mixes a saturated solution of NaCl and deionized water automatically in the appropriate proportions. The pH of the resulting solution is modulated varying the amount of the cathode product that is recirculated through the electrochemical cell. In all cases, the samples were taken when the machine was stable and used immediately after its generation or stored at 5 °C until use to ensure stability. The free available chlorine (FAC) was measured with hand‐held Colorimeter Chlorine UHR provided by Hanna Instruments (Woonsocket, RI, USA). pH, oxygen redox potential (ORP) and conductivity were measured using a pH/Ion/DO Multimeter SG68 (Mettler Toledo, Columbus, OH, Spain). The main characteristics of the EW products and their use in the different experiments are indicated in Table 2.

**Table 2:** Electrolyzed water products used in the experiments and their characteristics

	pH	FAC (ppm)	ORP (mV)	Conductivity (mS cm^−1^)	Type of study	Year
EW 1	5.7	490	1089	7.9	Mycelial growth/conidia germination	2021
EW 2	2.9	456	1122	7.8	Mycelial growth/conidia germination	2021
EW 3	5.7	152	1091	2.8	Mycelial growth/conidia germination	2021
EW 4*	5.3	502	1092	8.3	Nursery experiment/cuttings	2021
EW 5	4.5	107	904	1.0	Nursery experiment/cuttings	2023

Abbreviations: EW, electrolyzed water; FAC, free available chlorine; ORP, oxidation‐reduction potential.

* Sample was diluted 10‐fold with water before use.

### 2.3 Effect of EW on conidial germination

Fungal isolates of *C*. *luteo‐olivacea*, *D*. *torresensis*, *I*. *liriodendri*, *Pm*. *minimum* and *Pa*. *chlamydospora* were grown on potato dextrose agar (PDA) and incubated for 2–3 weeks at 25 °C in darkness. A conidial suspension was prepared for each isolate by flooding the agar surface with 10 mL sterile distilled water (SDW) and scraping with a sterile spatula. The resulting spore suspension was filtered through two layers of cheesecloth into a 250‐mL erlenmeyer flask. The filtrate was diluted with SDW and conidial concentration was adjusted with a hemacytometer to 10^6^ conidia mL^−1^.

The methodology to determine the effect of EW on conidial germination was adapted from Gramaje *et al*.^30^ and Di Marco *et al*.^29^ Fifty microliters of conidial suspensions were mixed with 950 μL each EW product (EW1, EW2 and EW3), using a vortex in 20‐mL glass tubes for 15, 30 or 60 s. Exposure was stopped by adding 9 mL neutralizing buffer (43 ppm monopotassium phosphate and 160 ppm sodium thiosulphate prepared in deionized water) at pH 7.2. Controls were prepared mixing 50 μL conidial suspensions with 950 μL of SDW and adding 9 mL neutralizing buffer.

After the treatment with EW, 20‐μL drops of conidial suspensions were transferred to 1.5% water agar (WA) Petri dishes, which were incubated in the dark at 25 °C for 48 h and observed under light microscopy. The viability of conidia was assessed by counting the number of conidia out of 100 randomly selected per drop that had germinated at each assessment time. A conidium was considered as germinated if the length of the primary germ tube was equal to at least the length of a conidium. There were three replicates per product/isolate/time of exposure combination, and four drops per replicate were plated on WA. The experiment was repeated.

### 2.4 Effect of EW on mycelium survival

The methodology to determine the effect of EW on mycelium survival was adapted from Gramaje *et al*.^30^ Mycelium colonized agar plugs, 6 mm diameter, were cut from the growing edge of 2–3‐week‐old colonies of *B*. *dothidea*, *D*. *torresensis*, *E*. *lata*, *I*. *liriodendri*, *L*. *theobromae*, *N*. *parvum*, *Pm*. *minimum* and *Pa*. *chlamydospora* growing on PDA. Four agar plugs per isolate were placed into 10‐mL glass tubes containing 2 mL each EW product (EW1, EW2 and EW3). The tubes were shaken with a vortex for the following times of exposure 30 s, and 1, 5, 15 and 30 min. Immediately after EW treatment, agar plugs were removed from the tubes and blotted briefly, agar side down, on sterile filter paper (Whatman no. 2). Then, the agar plugs were washed by introducing them in 20‐mL glass tubes containing 10 mL SDW, which were shaken for 30 s with a vortex, and agar plugs were dried again on sterile filter paper. Viability of fungal mycelium of each isolate was evaluated placing the treated plugs in the center of PDA plates supplemented with 0.5 g L^−1^ streptomycin sulfate (Sigma‐Aldrich) (PDAS) and incubated at 25 °C in darkness for 10 days. Additionally, four plugs of each isolate were placed in the center of four PDAS plates to serve as a control treatment. An agar plug was considered viable if a fungal colony was growing from it. There were three replicates per product/isolate / time of exposure combination and the experiment was repeated twice.

### 2.5 Nursery experiments

The experiments were carried out in a nursery located in Fontanars dels Alforins, Valencia province (eastern Spain) in two different years: 2021 and 2023, using the products EW4 diluted at 10%, and EW5, respectively (Table 2).

**Figure 1:**
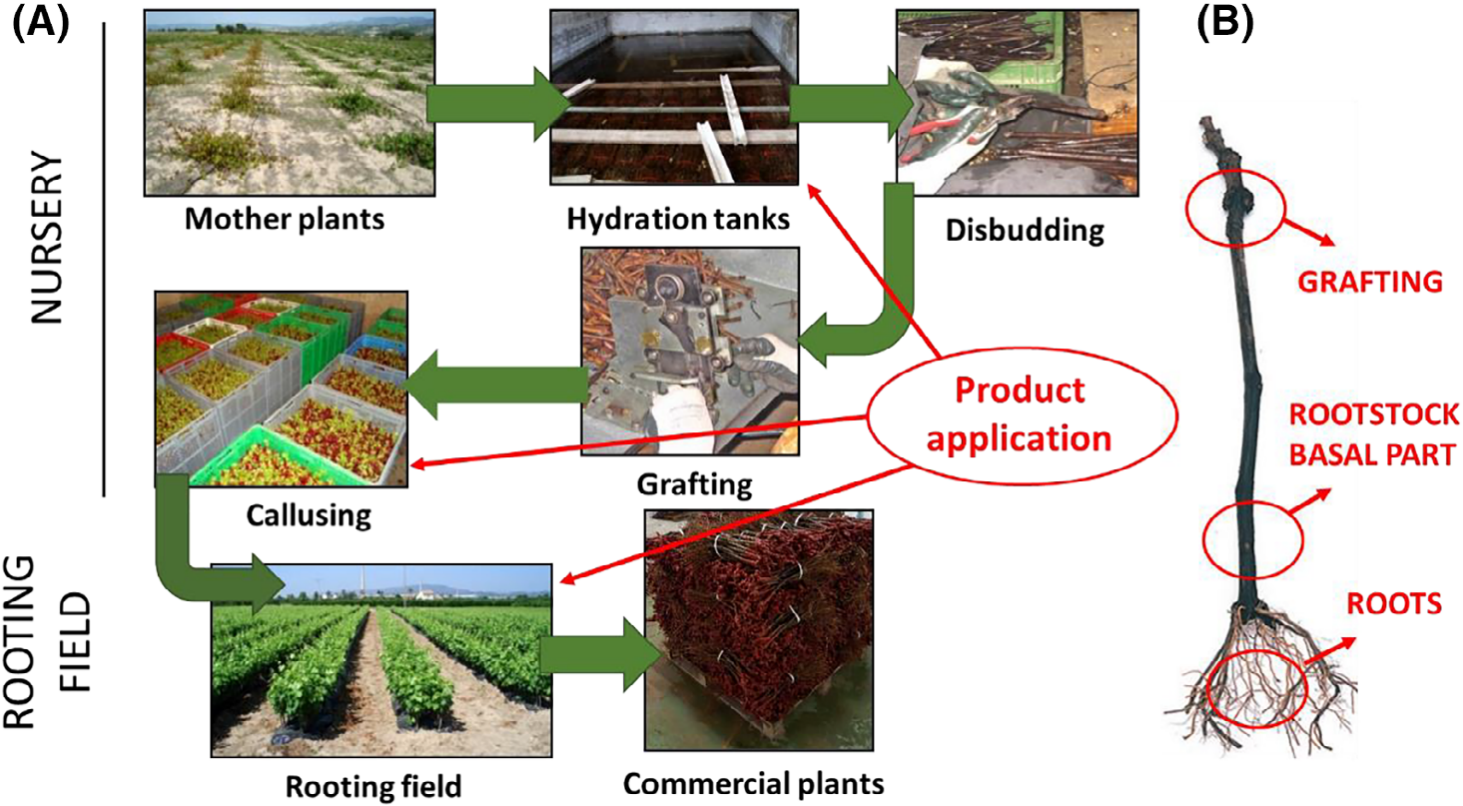
(A) Schematic representation of the EW treatments at three stages during the grapevine propagating process. The untreated control involved treatments with water at each of the three stages. Grafted plants were planted in a nursery rooting field in May and were arranged in a randomized complete block design with four replicates (100 plants) per treatment. (B) Isolations from plants were made from 1‐cm‐long sections cut from three different areas: the grafting point, the basal end of the rootstock cuttings, and the root system. EW, electrolyzed water.

Grapevine propagating material (cuttings of 110 R rootstock subsequently grafted with Macabeo cultivar for 2021 and rootstock 140 R/Macabeo for 2023) were treated with EW at three stages during the grapevine propagating process [Fig. 1(A)]: (i) a 24‐h soak in EW before grafting; (ii) the application EW by watering the sawdust at stratification; and (iii) a 1‐h soak of the basal parts of the plants in EW before planting in the rooting field. The untreated control involved treatments with water at each of the three stages. For each of the two treatments, EW and the untreated controls, there were four replicates of 100 grafted plants, which were managed separately.

Grafted plants were planted in a nursery rooting field in May and were arranged in a randomized complete block design with four replicates (100 plants) per treatment. Cultural practices were performed according to the common integrated pest management (IPM) guidelines for grapevine nurseries and only copper compounds and wettable sulfur were applied at label dosages to control downy and powdery mildew, respectively, if required.

Grafted plants were uprooted in October and wrapped in individual perforated plastic bags not only to avoid cross‐contamination, but also to prevent oxygen deprivation and fermentation, without exposing the cuttings to dehydration. Then, 30 grafted plants per treatment and replicate were selected randomly and taken to the laboratory for fungal isolation analyses. Isolations were made from 1‐cm‐long sections cut from three different areas: the grafting point, the basal end of the rootstock cuttings, and the root system [Fig. 1(B)]. These sections were washed under running tap water, surface‐disinfested for 1 min in a 1.5% sodium hypochlorite solution and washed twice with sterile distilled water. Then, five internal wood fragments per section were placed on malt extract agar (MEA) supplemented with 0.5 g L^−1^ streptomycin sulfate (MEAS) (seven fragments per Petri dish, 21 wood fragments per plant). Plates were incubated for 10–15 days at 25 °C in the dark, and all emerging colonies were transferred to PDA. Preliminary morphological identification of the colonies was conducted by observation of cultural and microscope characters for *Botryosphaeriaceae*, *C*. *luteo‐olivacea*, *Cylindrocarpon*‐like asexual morphs, the genus *Phaeoacremonium* and *Pa. chlamydospora*.^31^, ^32^, ^33^, ^34^, ^35^, ^36^

For species identity confirmation, fungal mycelium and conidia from pure cultures grown on PDA for 2–3 weeks at 25 °C in the dark were scraped and mechanically disrupted using FastPrep‐24™5G (MP Biomedicals, Santa Ana, CA, USA). Total DNA was extracted using the E.Z.N.A. Plant Miniprep Kit (Omega Bio‐tek, Doraville, GA, USA) following the manufacturer's instructions. The quality and integrity of the DNA was visualized on 1% agarose gels stained with Realsafe (Durviz S.L., Valencia, Spain). All DNA samples were stored at −20 °C. The identification of all isolates was performed by analysis of the internal transcribed spacer (ITS) region amplified using the fungal universal primers ITS1F and ITS4.^37^, ^38^ Then, further molecular identification was conducted for specific groups of pathogens. *C*. *luteo‐olivacea* and *Phaeoacremonium* species were identified by sequence analysis of the β‐tubulin gene. For *C*. *luteo‐olivacea* the primers used were BTCadF and BTCadR,^39^ and for *Phaeoacremonium* they were T1 and Bt2b.^40^, ^41^ Identification of Botryosphaeriaceae species was confirmed by analysis of elongation factor 1‐α gene amplified using EF1F and EF2R primers.^42^ Identification of *Cylindrocarpon*‐like asexual morphs was confirmed by sequencing part of the histone H3 gene with primers CYLH3F and CYLH3R.^43^

During the 24 h of cuttings soaking before grafting, the evolution of Cl_2_, pH, conductivity and ORP was studied as a function of time. Likewise, dynamics of weight gain of the sticks in the immersion process was studied as a proxy to estimate the penetration of the product in the wood.

### 2.6 Statistical analysis

For the *in vitro* experiments, values of conidia germination inhibition relative to the nontreated control for the different products and exposure times were calculated as mean percentages resulting from the three replicates including four drops of conidia suspensions each. Values of mycelial disc survival for the different products and exposure times were calculated as mean percentages resulting the three replicates including four discs each.

In each nursery experiment, the number of infected plants per treatment replicate was estimated based on the positive isolations from the grafted plants of FTPs associated with Petri disease (*C*. *luteo‐olivacea*, *Phaeoacremonium* spp. and *Pa*. *chlamydospora*), belonging to the family Botryosphaeriaceae for Botryosphaeria dieback, and *Cylindrocarpon*‐like asexual morphs for black‐foot disease. Disease incidence was expressed as the mean percentage of infected plants.

Statistical analyses were conducted using R v4.2.0.^44^ For treatment effect, values were analyzed using the Kruskal–Wallis multiple comparison test. When differences in the means were significant, Dunn's *post hoc* test was applied using the packages agricolae’ ^45^ and dunn.test.

## 3 RESULTS

### 3.1 Effect of EW on conidial germination

**Figure 2:**
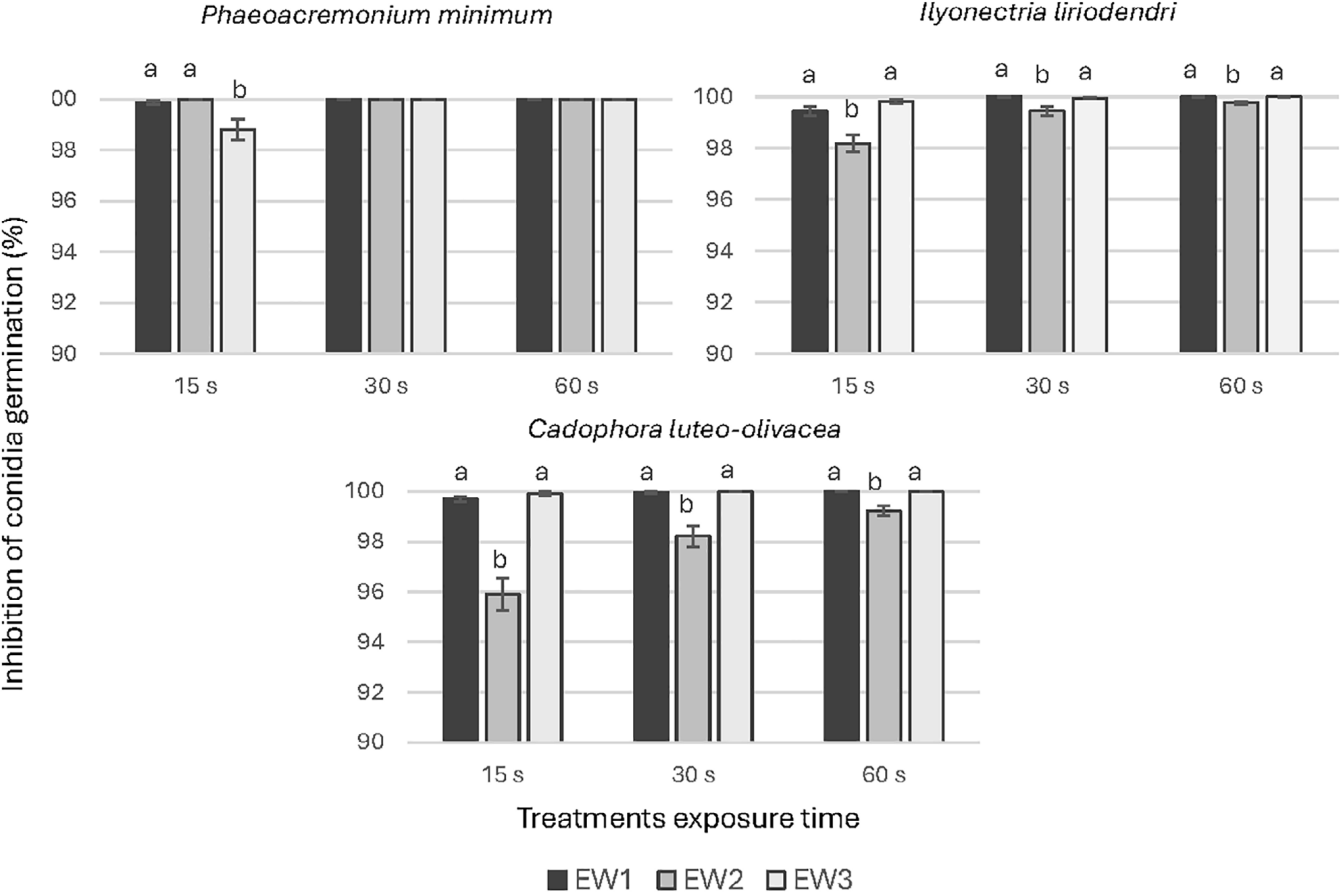
Percentage of conidia germination inhibition relative to the control for the different products EW1, EW2 and EW3, and exposure times (15, 30 and 60 s). Data are the mean values resulting from the three replicates including four drops of conidia suspensions each. Same letters represent no significant differences (*P* < 0.05) among the products for each exposure time and bars are standard errors. EW, electrolyzed water.

Products EW1, EW2 and EW3 (Table 2) were used in this study. Thus, we were able to evaluate the effect of diverse pH and FAC. Despite the differences among the products all of them showed >95% conidia germination inhibition for all fungal pathogens evaluated after 15 s exposure. In the case of *D*. *torresensis* and *Pa*. *chlamydospora* no significant differences were observed for the treatments, with all of the products showing >99.4% of germination inhibition after 15 s. Slightly variable responses were observed for the other three fungal pathogens evaluated, whose germination percentages are shown in Fig. 2. For the species *I*. *liriodendri* and *C*. *luteo‐olivacea* significant differences were observed among products. The products at pH 5.7 showed higher inhibition relative to the control for all treatments and exposure times than at pH 2.9 (Fig. 2). Furthermore, for *Pm*. *minimum* a significant difference among the products was observed only in the 15‐s exposure time treatment, being EW3 less efficient for the inhibition of conidia germination than EW1 and EW2 (Fig. 2). Higher exposure time did not increase EW inhibition of conidial germination in the case of EW1 or EW3, but increased the effectivity of the acid product (EW2).

### 3.2 Effect of EW on mycelium survival

**Figure 3:**
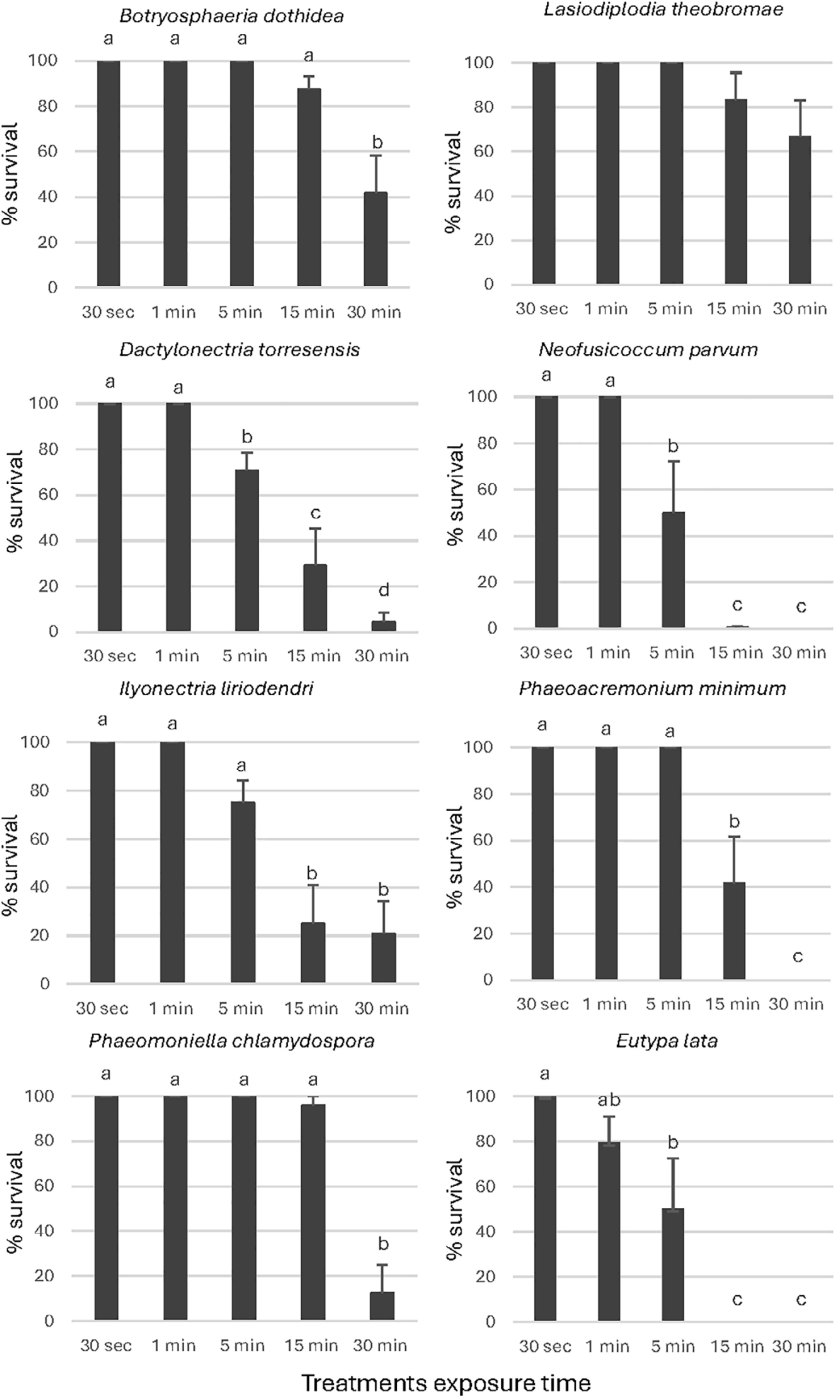
Percentage of mycelial discs survival using the product EW1 for the treatments. Data are the mean values resulting from the three replicates including four discs each. Same letters represent no significant differences (*P* < 0.05) among the products for each exposure time and bars are standard errors. EW, electrolyzed water.

**Figure 4:**
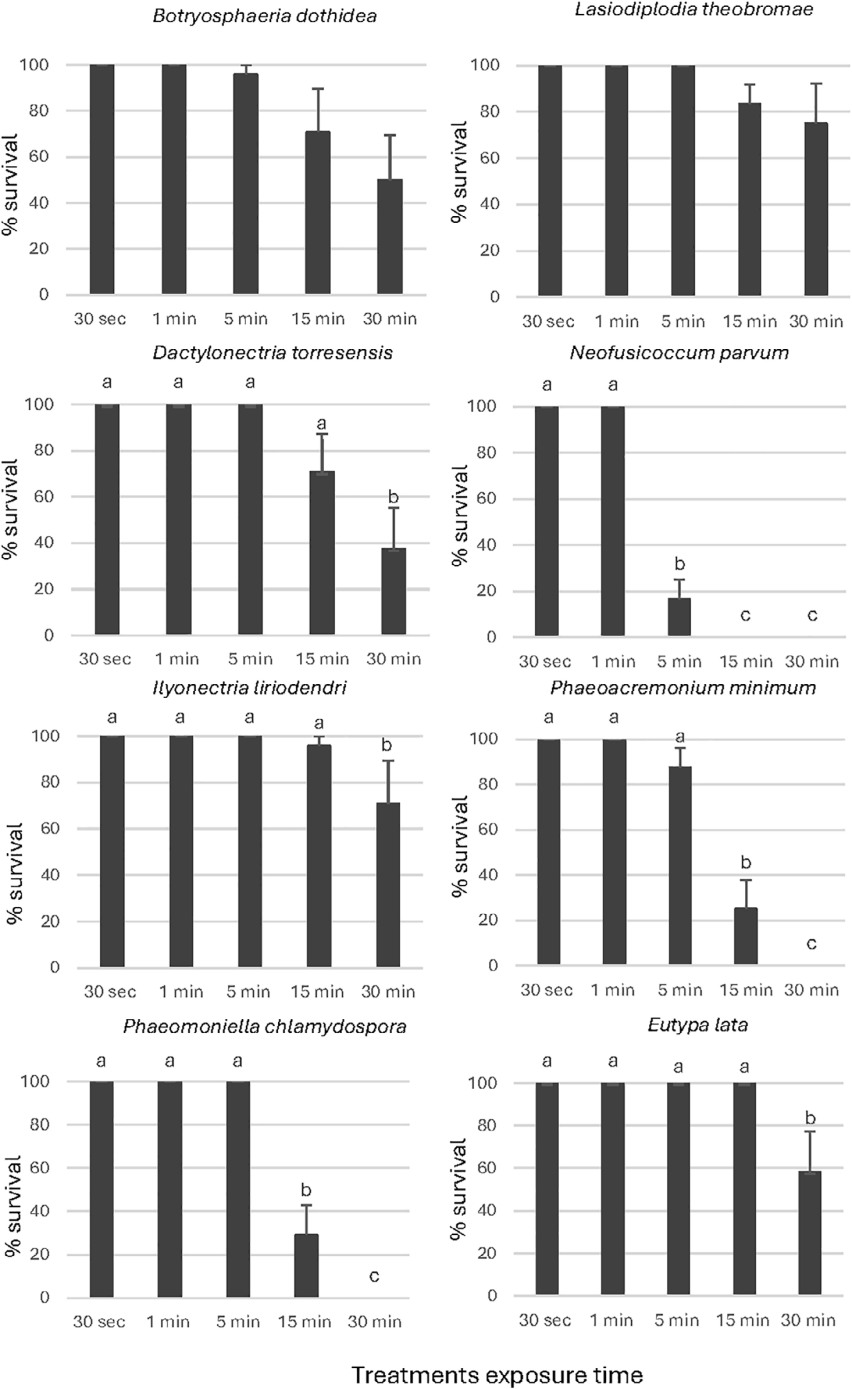
Percentage of mycelial discs survival using the product EW2 for the treatments. Data are the mean values resulting from the three replicates including four discs each. Same letters represent no significant differences (*P* < 0.05) among the products for each exposure time and bars are standard errors. EW, electrolyzed water.

**Figure 5:**
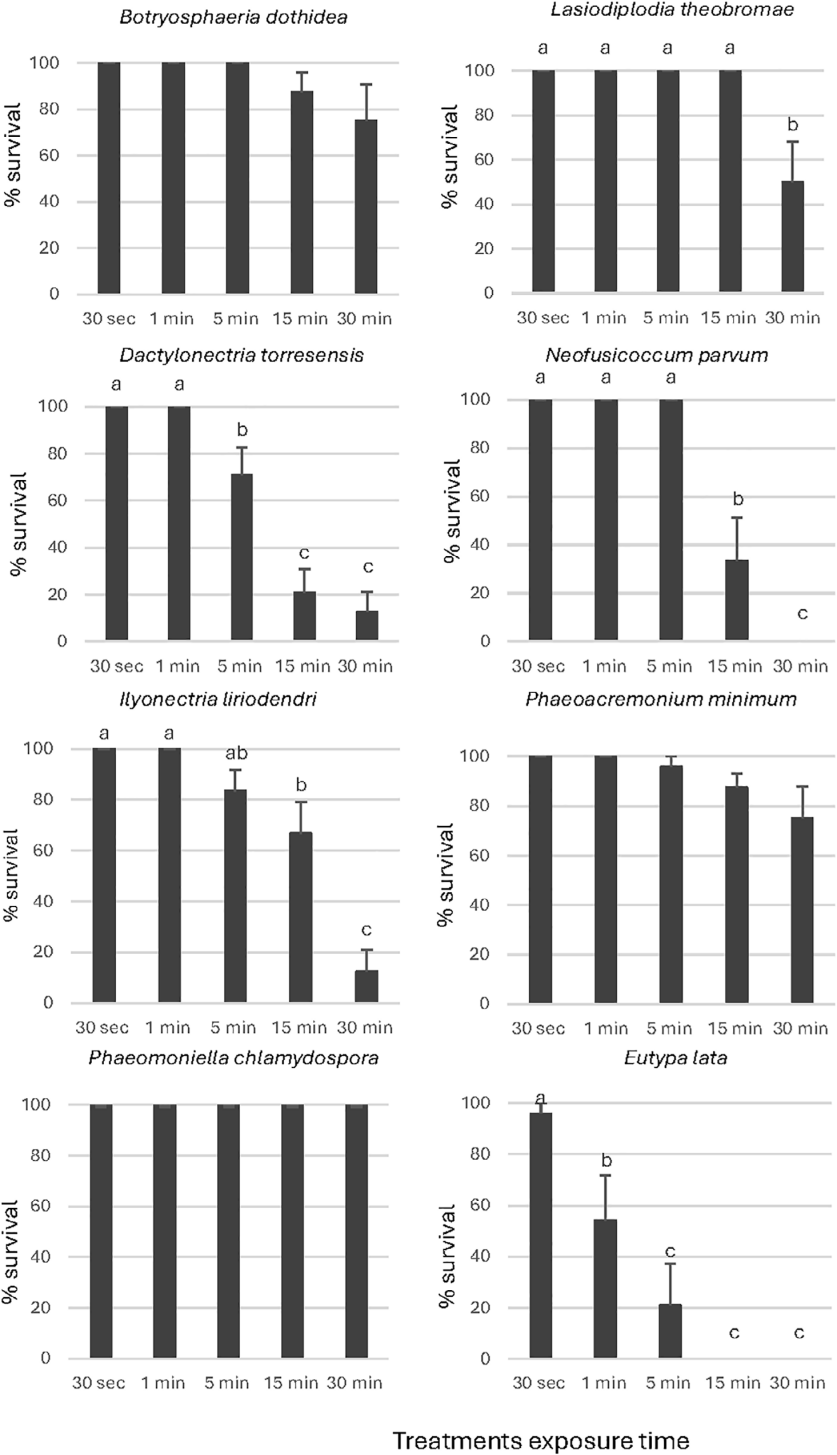
Percentage of mycelial discs survival using the product EW3 for the treatments. Data are the mean values resulting from the three replicates including four discs each. Same letters represent no significant differences (*P* < 0.05) among the products for each exposure time and bars are standard errors. EW, electrolyzed water.

For the mycelium survival (Figs 3, 4, 5), a significant effect of the exposure time was observed in the percentage of survival of *D*. *torresensis*, *E*. *lata*, *I*. *liriodendri* and *N*. *parvum* for all products evaluated. For *B*. *dothidea* and *L*. *theobromae* the mycelium survival decreased with time for all the exposure times and products; however, this difference was significant for *B*. *dothidea* with EW1, and *L*. *theobromae* with EW3 only. In the case of *Pm*. *minimum* and *Pa*. *chlamydospora*, only EW1 and EW2 showed a significant reduction in mycelium survival as treatment exposure time increased. According to our results, EW1 was the most effective because it showed a significant reduction in mycelium survival with the treatment's exposure time for all of the fungal pathogens evaluated except for *L*. *theobromae*.

*Dactylonectria torresensis* and *N*. *parvum* showed high sensitivity to EW1, with a significant reduction of survival percentage after 5 min of exposure (Fig. 3). For this product *I*. *liriodendri*, *Pm*. *minimum* and *E*. *lata* mycelial growth was significantly reduced after 15 min of exposure and 30 min in the case of *B*. *dothidea* and *Pa*. *chlamydospora* (Fig. 3).

EW2 treatments significantly reduced mycelium survival of *N*. *parvum* after 5 min of exposure (Fig. 4). Mycelium survival of *Pm*. *minimum* and *Pa*. *chlamydospora* was significantly reduced after 15 min of exposure and *D*. *torresensis*, *I*. *liriodendri* and *E*. *lata* mycelium survival significantly decreased after 30 min (Fig. 4).

Results for EW3 demonstrated that *E*. *lata* and *D*. *torresensis* were the most sensitive species. showing a significant mycelium survival reduction after just 1 and 5 min of exposure, respectively (Fig. 5). Mycelial survival of *Pm*. *minimum* and *Pa*. *chlamydospora* was significantly reduced after 15 min of exposure and *L*. *theobromae* mycelium survival significantly decreased after 30 min (Fig. 5).

### 3.3 Nursery experiments

In both nursery experiments, there were no negative effects of the treatments regarding the viability of the grafted plants. The isolation of FTPs was highly variable. Isolation data from the different FTPs found were grouped according to the three main diseases considered: Petri disease (including *C*. *luteo‐olivacea*, *Pm*. *minimum* and *Pa*. *chlamydospora*): Botryosphaeria dieback (including fungal isolates belonging to the family Botryosphaeriaceae); and black‐foot disease (including *D*. *torresensis* and *I*. *liriodendri*) and used to calculate their incidence (mean percentage of infected plants).

**Figure 6:**
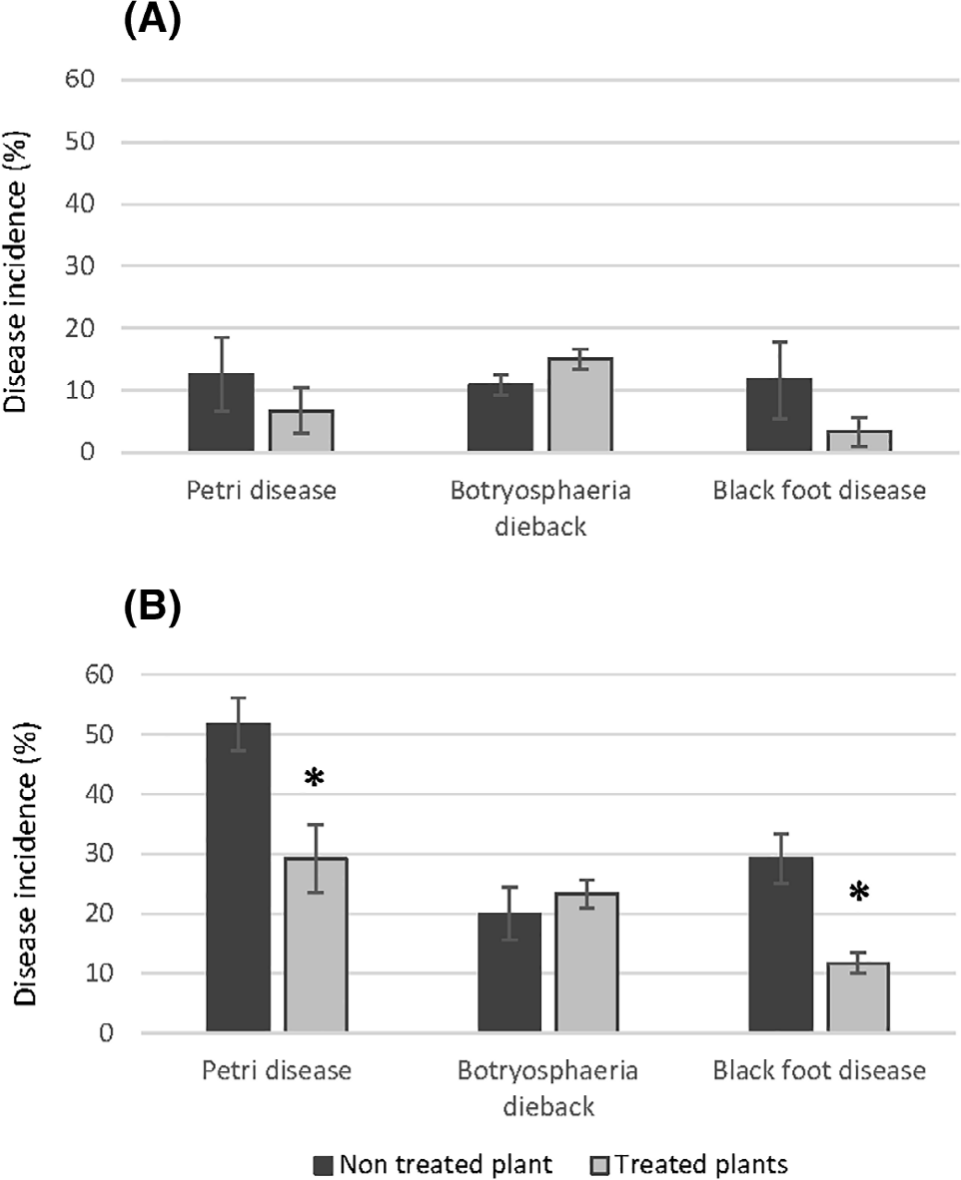
Disease incidence (percentage of plants infected by pathogens associated with Petri and black‐foot diseases and Botryosphaeria dieback) in grapevine nursery experiments across two growing seasons, 2021 (A) and 2023 (B). Values are the mean of four replicates of 30 plants and vertical bars are the standard errors of the mean. Significant differences between treatment and untreated control plants (*P* < 0.05) are indicated with asterisks.

Significant differences were observed between EW4 and EW5 (*P* = 0.01). In general, disease incidence values were lower in the first experiment compared with the second, with values ranging from 3.5 to 15 and from 11.6 to 51 in 2021 (EW4) and 2023 (EW5), respectively (Fig. 6). In 2021, using EW4, percentage of plants infected by pathogens associated with Petri and black‐foot diseases showed a reduction with the treatments, but no significant differences were observed in disease incidence between treated and nontreated plants [Fig. 6(A)]. By contrast, in 2023, with a solution of higher FAC, results showed a significant (*P* < 0.05) reduction for the same disease incidence [Fig. 6(B)].

**Figure 7:**
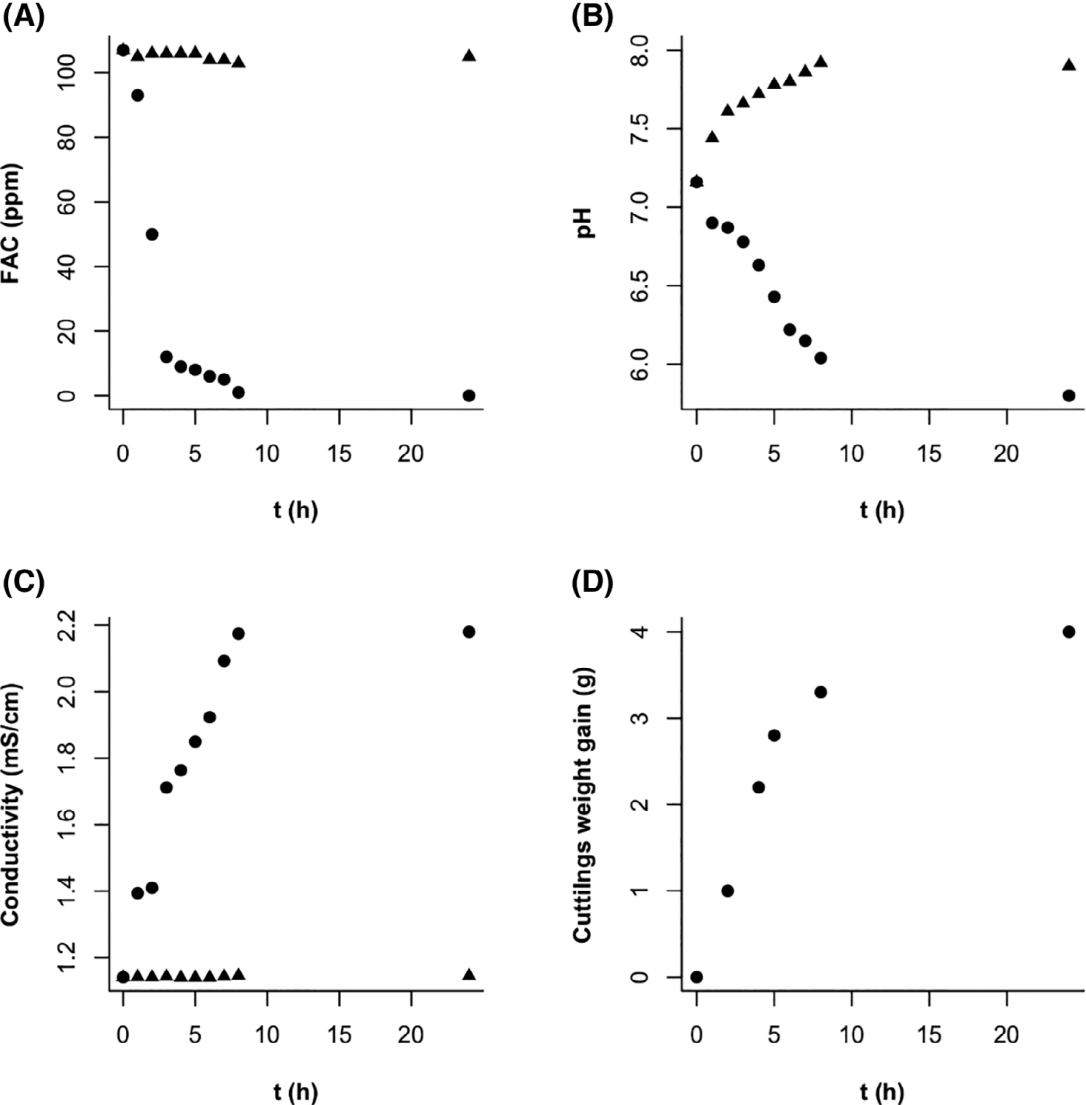
Evolution of the EW bath and the cuttings during a 24 h soak (⚫), or EW in absence of cuttings (▲). (A) Free available chlorine (FAC), (B) pH and (C) conductivity measured in the immersion batch. (D) Weight gain of the cuttings during the immersion. EW, electrolyzed water.

In 2023 the influence of the grapevine propagating material in the main properties of EW5 [Fig. 7(A)–(C)], and the evolution of the weight gain as an indication of the hydrating process [Fig. 7(D)] were studied. FAC decreased very fast in the first 2 hours, reaching a value <20 ppm. However, the interaction between the cuttings and the solutions continued for 6 h more, as indicated by the changes in conductivity and pH, and weight gain. These changes could be assigned to the cuttings because the control (EW5 in absence of cuttings) did not suffer strong variations in the key parameters.

## 4 DISCUSSION

In our research, the effect of EW against a wide range of FTPs affecting grapevine was evaluated in both laboratory‐controlled and nursery conditions. Initial *in vitro* experiments demonstrated the capacity of different EW products to reduce conidial germination and mycelium survival of selected FTPs belonging to different genera and species, although the results were variable depending on the type of product, pathogen evaluated and time of treatment.

A previous study conducted by Di Marco *et al*.,^29^ showed a consistent decrease in conidial germination of the FTPs *Pm*. *minimum* and *Pa*. *chlamydospora* exposed to an EW product with 40 ppm Cl and pH = 2.5, but no significant reduction of *in vitro* mycelial growth. Our results regarding conidial germination of both pathogens agree with those of Di Marco *et al*.^29^ and we observed that higher exposure time increased the effectivity of the acid product. EW, which contains HOCl, exhibits greater stability and antimicrobial efficacy under acidic conditions. This is because HOCl is more prevalent at lower pH levels, where it remains chemically stable and can more effectively permeate microbial cell membranes. Furthermore, acidic solutions exhibit a higher ORP, enhancing the disinfectant properties of EW by facilitating oxidative damage to microorganisms.^46^

We also obtained significant reductions of mycelium survival when the time of treatment increased. It is interesting to note that for conidial germination we used the same methodology described by Di Marco *et al*.,^29^ but for mycelium survival we adapted a methodology used previously to evaluate the effect of hot‐water treatments on FTPs,^30^, ^47^, ^48^ in which the mycelium plugs were immersed into the EW products and stirring ensured a good contact between fungal mycelium and EW.

Our results confirmed a very quick effect of EW as indicated by Di Marco *et al*.^29^ A few seconds of exposure of fungal conidia to EW were sufficient to significantly reduce conidial germination of different FTPs. Moreover, a significant reduction also was observed for mycelium survival, although this effect was noticeable with exposure times >15 and 30 min. Therefore, the activity of EW against FTPs fits perfectly with the needs for disinfection of grapevine grafted plants in the nursery production process, especially in the initial hydration stage of the plant material before grafting. In this phase, following cold storage, rootstock and scion cuttings are usually soaked in water for periods of 4 h to 4 days,^2^, ^7^ which according to our results is a time period well above the minimum required for EW‐based treatments to be effective. The application of EW products can contribute to reduce FTP inoculum from water used for soaking cuttings, the presence of which has been reported in grapevine nurseries in many countries.^7^ Gramaje *et al*.^49^ demonstrated that the species *Pm*. *minimum* and *Pa*. *chlamydospora* can infect healthy cuttings during the hydration stage, suggesting that mycelium and conidia present on the surfaces of cuttings might wash off into the water during hydration, or it might even ooze from xylem vessels into the water. Thus, hydration tanks containing drenches for soaking are an important focus for FTP management strategies.^2^, ^7^, ^49^

Nursery experiments were conducted to assess the potential of EW treatments to reduce infections caused by FTPs on propagation material. These experiments were performed following protocols similar to those used in previous studies aiming to determine the effect of biological control agents (BCA) to control FTPs during the grapevine propagation process.^50^, ^51^ This approach could facilitate the comparison of the results between different management strategies. The EW was applied at three stages of the propagation process: hydration, stratification, and before planting in the rooting field.^50^, ^51^ Then, the reduction of the wood infections was assessed at the end of the nursery propagation process, on vines ready to be planted in the vineyard in spring after winter cold storage. Moreover, in agreement with the *in vitro* results, EW products with a pH ≈5, but with a lower FAC, were selected for the nursery experiments to maintain the fungicidal activity but avoid the risks of phytotoxicity. For this reason, EW4 was produced at 502 ppm Cl and then, it was diluted to 10% to achieve a lower free Cl concentration. For the second nursery trial EW5 at 100 ppm was used, leading to better disinfection outcomes. For this treatment at the second nursery trial a pH value of 4.5 was chosen because the product is more stable at an acidic pH, but lower pH values can potentially have harmful effects for the cuttings. A balance between pH and Cl concentration is needed in order to prevent detrimental adverse effects on the grapevine cuttings resulting from very low pH values or high Cl concentration, which have been reported in other crops.^52^ In fact, di Marco *et al* observed that prolonged immersion of grapevine cuttings in EW caused a slight discoloration of the wood surface.

In the two nursery experiments, treated plants showed lower incidence of Petri and black‐foot diseases when compared with the untreated ones, although differences relative to the control were statistically significant only in 2023. Actually, the highest incidence of FTPs on untreated control cuttings was observed in 2023. However, EW did not show any reduction effect on the incidence of Botryosphaeria dieback. Airborne conidia and/or ascospores of Botryosphaeria dieback fungi are dispersed during rain events or under moist conditions, and infection of grapevine tissues can occur mainly through pruning wounds or weak graft unions.^4^, ^53^ Thus, we can hypothesize that most infections caused by these pathogens were produced in the nursery field after the EW treatments had been applied. On the contrary, in previous nursery studies the application of BCA such as *Trichoderma atroviride* SC1 alone or combined with *Bacillus subtilis* PTA‐271 was able to significantly reduce the incidence of Botryosphaeria dieback fungi on nursery grafted plants,^50^, ^51^ probably owing to the capacity of the BCA to colonize grapevine tissues, providing a long‐term protection on any wound in the aerial part of the grafted cuttings. For instance, *T*. *atroviride* SC1 showed high levels of reisolation from all treated plants at the end of the nursery experiments.^50^, ^51^

Regarding Petri and black‐foot diseases, our results showed that the application of EW during the propagation process can reduce infections caused by fungal pathogens associated with these diseases. It is well‐known that the grafting process increases the risk of contamination by Petri and black‐foot pathogens, being the rooting phase in nursery fields in which the infections are most likely to occur.^2^ In particular, many studies have emphasized that bhlack‐foot pathogens are very difficult to control because of their soilborne nature, and the abundant wounds that occur on grapevine cuttings during the grapevine propagation process, callus formation and root development.^35^ Several studies have already reported the low effectiveness of *Trichoderma* spp. against Black‐foot associated pathogens.^50^, ^54^, ^55^, ^56^ Therefore, EW could be a suitable treatment for grapevine nurseries in those regions in which black‐foot disease is one of the main constraints when establishing new vineyards.

Another important finding in the nursery experiment was the behavior of EW when absorbed by the cuttings during the 24‐h soaking period. The main interaction of the cuttings with the EW was observed during the first 8 h of soaking as revealed by the gained weight and the main parameters of the solution registered over time. There was a first step up to 3 hours in which FAC was high. According to the *in vitro* results, this time was sufficiently long to guarantee sufficient biocide activity. However, the EW reaction with the organic matter of the cutting reducing FAC might result in 3–7 h of relatively low biocide concentration and negligible concentration after 8 h, beyond which no fungicidal effect would be expected. Cutting weight gain would be explained by continuous water absorption for ≤5 h. However, the chemical exchange between the grape wood and the EW solution would induce an increase of conductivity and pH reduction ≤8 h in agreement with a salt extraction from the cuttings and a wood‐induced slight acidification.

## 5 CONCLUSION

Chemical control of FTPs with fungicides has been explored as an efficient strategy to prevent or reduce fungal infection of grapevine propagation material, but few active ingredients have shown a wide spectrum activity, being able to control the huge diversity of taxonomically unrelated FTPs able to infect grapevines.^49^, ^57^ EW is an alternative treatment offering a wide range of antimicrobial efficacy. In our study, *in vitro* evaluation of different EW products demonstrated their capacity to reduce conidial germination and mycelium survival of relevant FTPs belonging to different genera and species at very low exposure times. This confirms that EW treatments can be useful for the disinfection of grapevine planting materials in the nursery production process, especially in the initial hydration stage of the plant material before grafting. Moreover, EW treatments applied in a grapevine nursery reduced the incidence of Petri and black‐foot pathogens in grafted cuttings when compared with the untreated ones. EW treatments could be integrated with other complementary IPM strategies and also be extended to nurseries of other fruit and nut crops in which FTPs are currently becoming important emerging diseases, that require the use of planting material of the highest phytosanitary quality.

## Data Availability

The data that support the findings of this study are available on request from the corresponding author. The data are not publicly available due to privacy or ethical restrictions.
